# Evaluation of the immune response of dogs after a mass vaccination campaign against rabies in Tunisia

**DOI:** 10.1186/s12917-023-03582-8

**Published:** 2023-01-30

**Authors:** Mariem Handous, Imed Turki, Abdejelil Ghram, Samia BenMaiz, Jihen Bensalem, Nourhene Basdouri, Mohamed Soltani, Farah Bassalah, Habib Kharmachi

**Affiliations:** 1 University of Tunis El Manar, Rabies Laboratory, Institut Pasteur of Tunis, Tunis, Tunisia; 2 Contagious Diseases, Zoonoses and Health Legislation Department, University of Manouba, National School of Veterinary Medicine, Sidi Thabet, Tunisia; 3 University of Tunis El Manar, Laboratory of Epidemiology and Veterinary Microbiology, Institut Pasteur of Tunis, Tunis, Tunisia

**Keywords:** Rabies virus, Dogs, Immune response, FAVN

## Abstract

**Background:**

Rabies (RABV) is an enzootic disease in Tunisia, with dogs being the primary reservoir. Vaccinating dogs is the key to eradicate rabies. Regional Veterinary Services conduct nationwide immunisation campaigns on an annual basis. Evaluation of the immune response is still important to make sure that the vaccination is effective in the conditions of the Tunisian field. In this paper, the FAVN technique was used to test rabies antibody dynamics in dogs from three distinct Tunisian areas observed for one year following a mass vaccination campaign.

**Results:**

On day 30 after vaccination, 75% of all dogs vaccinated during the campaign were sero-positive (titres greater than or equal to 0.5 transformed IU/ml). On day 180, 48% of all dogs were sero-positive. Only 25.6% of primary-vaccinated dogs remained sero-positive on day 180 and 7% on day 365, whereas 91% of previously sero-positive dogs remained sero-positive on day 365.

**Conclusions:**

Although a single rabies vaccine is successful at stimulating an immunological response, it is recommended that primary-vaccinated dogs have a second booster between one and three months after the initial vaccination to maintain seropositivity. To achieve the rabies eradication objective, all dogs should receive an annual booster to maintain effective immunological protection.

**Supplementary Information:**

The online version contains supplementary material available at 10.1186/s12917-023-03582-8.

## Background

Rabies (RABV) is a fatal enzootic and endemic disease that is present in over 150 countries, mostly in Africa and Asia where the main reservoir is the dog. The majority of its victims are children [1]. It kills one person every nine minutes. Despite the fact that a vaccine has been available since 1885, the vaccination rate for dogs does not meet the 70% vaccination threshold needed to eliminate the disease [2–7]. Frequent vaccination efforts are necessary [8] to maintain high levels of vaccine coverage.

In Tunisia, dogs remain the only reservoir of rabies [9]. Rabies vaccination has been mandatory for all dogs since 1982. However low vaccination rates have made rabies control programmes less effective [10]. Reported cases of rabies in dogs have more than doubled from 2010 to 2017, while vaccination rates have remained consistent [10, 11].

An antibody titre of 0.5 IU/ml is considered an effective immune response [12]. Vaccination effectiveness is also reliant on vaccine potency, which is crucial considering Tunisia's average annual temperature of 23 °C [13]. Even though vaccines are becoming more stable at relatively high temperatures and have proved their potential to induce an immune response [14], maintaining a reliable cold chain remains the goal [15, 16].

The objective of this study was to evaluate the dynamics of rabies antibody titres in dogs from the field vaccinated against rabies during mass vaccination campaigns in three diverse areas in Tunisia over one year. Findings will help veterinarians participating in dog vaccination campaigns to better understand dogs' immunological responses to rabies and, ultimately, to adapt the vaccination program for a higher and longer seropositivity rate of dogs.

## Results

### Characteristics of the studied dogs

Thirty two dogs were evaluated at all five time points (D0, D30, D90, D180, and D365), while 51 dogs were evaluated at four time points. The absence of owners, dog handlers, and the change in dog’s location caused a decline in the number of dogs reported.

Sixty four percent (64%) of dogs were males, whereas 36% were females. The age ranges of vaccinated dogs were 3–5 months (12%), 5–12 months (15%), and more than 12 months (73%).

In terms of size, 71% of dogs were large (over 20 kg), 13% were medium (between 20 and 10 kg), and 16% were small (below 10 kg).

In terms of nutrition, 43% of dogs received bread, 41% received leftovers, and 16% received meat and derivatives.

To avoid parasites, 5% of owners reported using a dewormer and/or a pest control product on their dogs, although we found ectoparasites in 18% of dogs on the initial visit in March 2017.

### Vaccination campaign features

All veterinarian facilities had a refrigerator, access to power, and employed traditional cool boxes with ice packs for onsite vaccination transfer. At least two people were on the immunisation team. Except for one site, the immunisation team used an all-terrain vehicle. Vehicles might be accessed at other facilities depending on how other activities were organised and only two facilities had a full-time vehicle available.

### Immune coverage

**Fig. 1:**
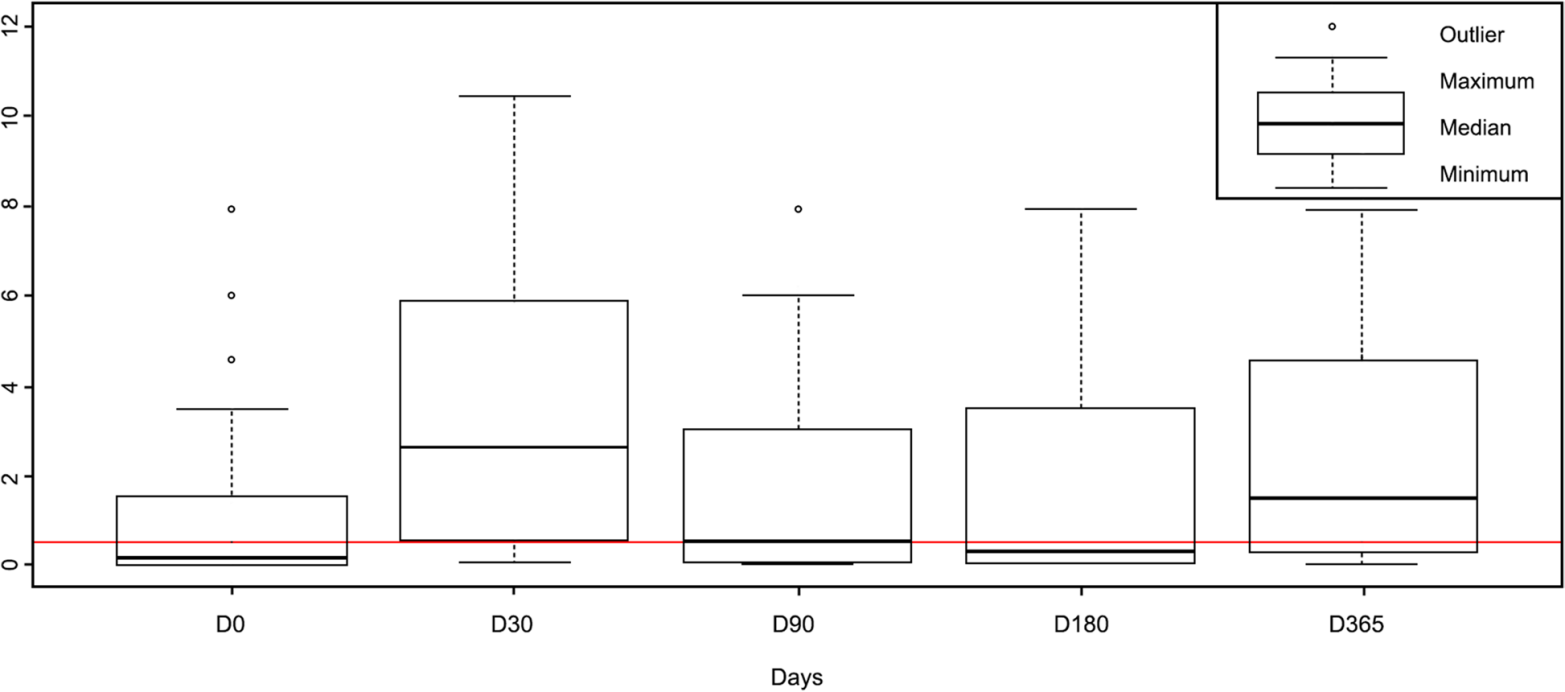
Rabies neutralising antibody titre dynamics in dogs after vaccination. The first quartile (lower dashed vertical lines), the interquartile range (box) and the third quartile (upper dashed vertical lines) are represented in this boxplot for each visit. The red line represents the seropositivity threshold (0.5 IU/ml). Abbreviations: IU/ml: International Units per millilitre

As shown in Fig. 1, the rabies antibody titres peaked at day 30 post-vaccination for all studied dogs, then declined on day 90 and continued to decline on day 180. The median titre on days 0, 30, 90, 180, and 365 was 0.13, 2.62, 0.50, 0.29, and 1.51 IU/ml, respectively.

### Factors of variation

**Fig. 2:**
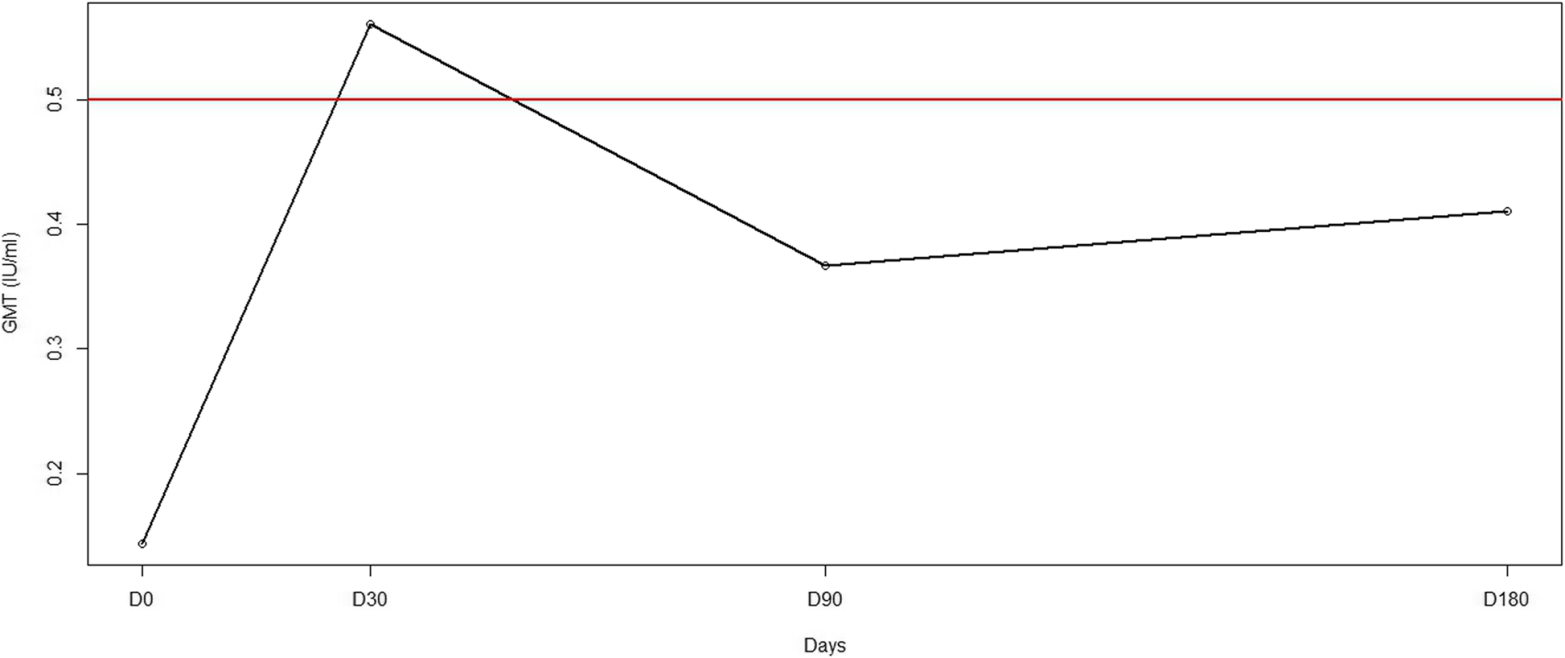
Rabies neutralising antibody Geometric Mean Titres in all studied dogs after vaccination. The red line represents the seropositivity threshold (0.5 IU/ml). Abbreviations: GMT: Geometric Mean Titres, IU/ml: International Units per millilitre

At each time point, GMTs (Geometric Mean Titres) were determined for all dogs. The calculated titre on days 0, 30, 80, 180, and 365 was 0.14, 0.56, 0.37, 0.41, and 3.86 IU/ml, respectively (Fig. 2). GMTs fell below the seropositivity threshold for all dogs between D90 and at least D180.

Due to the new mass vaccination effort that took place between D275 and D335, some dogs received a vaccination booster. To prevent a second seroconversion effect, D365 data were omitted from statistical analysis. We tested geometric means for logD50 values for statistical comparisons using the Shapiro test since their distribution was more normal (p: 0.025 vs 0.827).

For all the studied dogs, there were 35 sero-positive dogs out of 83 tested on D0, 58 out of 77 on D30, 47 out of 79 on D90, and 35 out of 73 on D180. Out of the original 35 sero-positive dogs on D0, 28 remained sero-positive on D30. Five dogs dropped below the seropositivity threshold despite being recently vaccinated and two dogs were unavailable for sample collection on this day, then returned for re-testing later in the study and maintained seropositivity through the remainder of the study. Among the 48 dogs sero-negative on D0, 30 dogs seroconverted on D30 after vaccination. Almost 85% of the sero-positive dogs on D0 were sero-positive on D30 and around 80% kept sero-positive on D180. In contrast, almost 69% of the sero-negative dogs on D0 were sero-positive on D30 but this percentage dropped to 26% on D180.

On D365, five previously sero-negative dogs seroconverted and became sero-positive after vaccination booster, with titres ranging from 0.5 to 4.56 IU/ml.

**Fig. 3:**
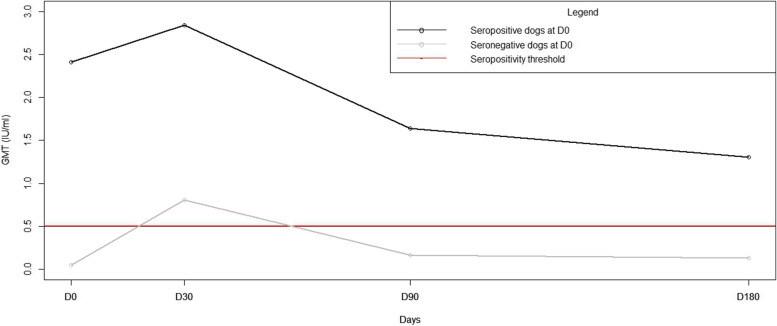
Comparison of Rabies neutralising antibody Geometric Mean Titres dynamics in initially sero-positive and sero-negative dogs after vaccination. Abbreviations: GMT: Geometric Mean Titres, IU/ml: International Units per millilitre

Rabies neutralising antibody GMTs were statistically higher in previously sero-positive dogs on D0 than sero-negative dogs on D0 (Student test, *p* < 0.05), and the difference was even more evident (Student test, *p* < 0.001) on D90. A model of multivariate linear regression corroborated these results (ANOVA, *p* < 0.001). For previously sero-positive dogs (*n* = 35), GMTs reached 2.84 IU/ml on day 30 (Fig. 3) and remained over the seropositivity threshold on day 180 (1.31 IU/ml). For previously sero-negative dogs (*n* = 48), GMTs reached 0.81 IU/ml on day 30 and decreased below the sero-positive threshold by day 90 (0.16 IU/ml).

The presence of ectoparasites did not influence the immune response of dogs. GMTs were 1.26 IU/ml (*n* = 16) in dogs with ectoparasites compared to 1.19 IU/ml (*n* = 42) in dogs without ectoparasites (Student test, *p* = 0.42).

### Age

**Fig. 4:**
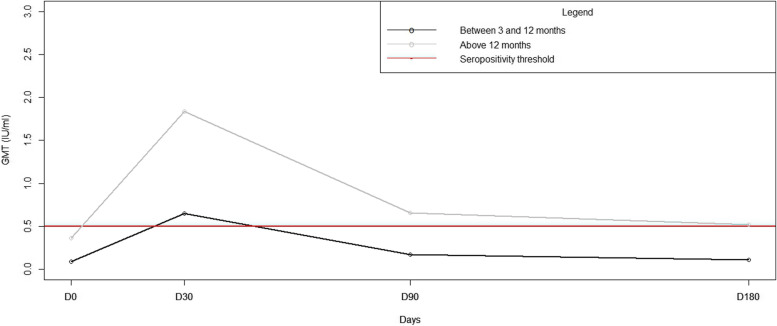
Comparison of Rabies neutralising antibody Geometric Mean Titres dynamics in young and elderly dogs after vaccination.Abbreviations: GMT: Geometric Mean Titres, IU/ml: International Units per millilitre

Comparing GMTs, we found that dogs over 12 months old (*n* = 57) exhibited considerably higher and long lasting titres (above seropositivity threshold, 0.65 IU/ml on D90 and 0.52 IU/ml on D180) than those between 3 and 12 months of age (*n* = 21) (0.17 IU/ml on D90 and 0.11 IU/ml on D180) (Fig. 4) (Student test, *p* = 0.001). A multivariate linear regression model confirmed these findings (ANOVA, *p* < 0.05).

We discovered that three dogs around 10 years old (inferred by loose teeth and arthritis) had high titres throughout the year (GMTs: 3.27, 6.01, and 6.35).

### Sex

For female dogs (*n* = 30) GMTs were 1.29 IU/ml on D30 and 0.49 IU/ml on D180. For male dogs (*n* = 52) GMTs were 1.41 IU/ml and 0.28 IU/ml for the same periods. There was no significant difference between the sexes (Student test, *p* = 0.76).

### Nutrition

Bread and leftovers fed dogs (*n* = 50) had a GMT of 1.42 IU/ml on D30 and 0.36 IU/ml on D180. Dogs (*n* = 8) given meat had GMTs of 1.64 and 0.54. No significant difference was found between the two groups (Student test, *p* = 0.67).

### Cold chain

**Fig. 5:**
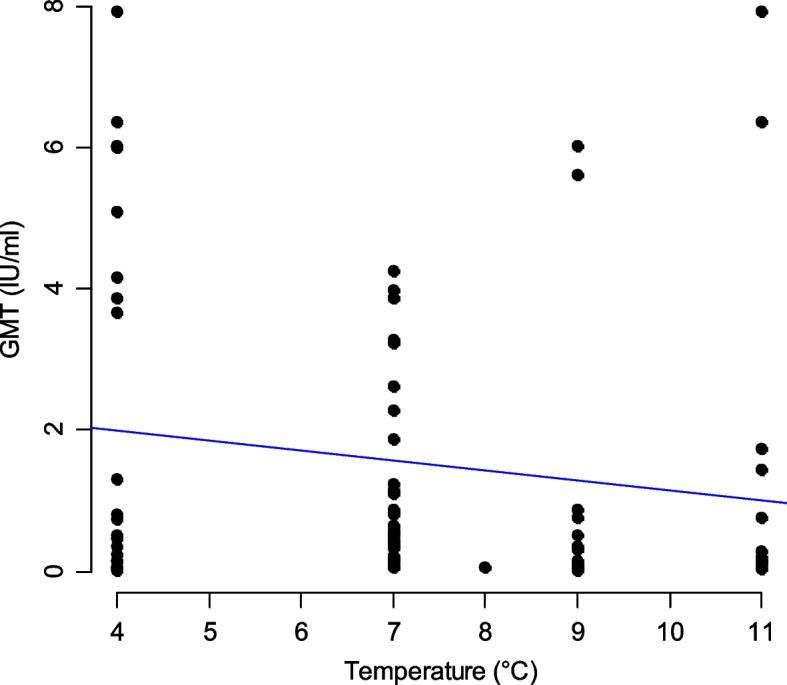
GMT scatterplot versus temperature recorded in coolers. Dots symbolise dogs individual GMTs. The blue line represents the trend line but it is not statistically significant. Abbreviations: °C: Celsius degrees, GMT: Geometric Mean Titres, IU/ml: International Units per milliliter

Mean temperatures recorded were 8.8 °C [7.1 – 11.5 °C] and 5.04 °C [2 – 7.9 °C] for the classical cool boxes and the refrigerated cooling devices respectively. Environmental temperature varied between 17 and 22 °C during the days the classical cool boxes were used and 15 and 21 °C during the days the refrigerated cooling devices were used. Vaccination sessions in the field lasted for about three hours on average [2–4 h].GMTs were 1.37 IU/ml (*n* = 46) for dogs immunised with vaccines conserved in classical cool boxes and 1.45 IU/ml (*n* = 37) for dogs immunised with vaccines conserved in refrigerated cooling devices. There was no significant difference between the two devices (Student test, *p* = 0.61) and no significant difference between GMTs and cooler temperature as shown in Fig. 5.

Even though the average maximum temperature in the classic cool box was 11.4 °C, one dog vaccinated on that day seroconverted and reached 4.56 IU/mL. On the day when we recorded the lowest average temperature in the refrigerated cooling devices (4 °C), we were able to monitor 14 dogs. Except one adult female, these fourteen dogs met the sero-positive threshold on D30. Seven of these 14 dogs were sero-negative at D0 but sero-positive at D90.

## Discussion

Although yearly vaccination campaigns have been undertaken since the 1980s, no recent studies have been published assessing their efficacy. Only two similar studies on vaccination monitoring have been published in Tunisia, and a comparative study was undertaken in Morocco in 2015. Furthermore, this is the first research in the country employing FAVN technique.

Haddad et al. [17] previously utilised a mouse seroneutralisation experiment to titrate dog sera comparing the effectiveness of a local vaccine, Rabirabta (Veterinary Research Institute, Tunisia), with a commercial vaccine, Rabisin (Merial, France). Later, Seghaier et al. [18] utilised RFFIT (Rapid Fluorescent Focus Inhibition Test) to titrate the sera of Rabirabta-vaccinated dogs. These prior studies demonstrated very similar dynamics to the present findings, albeit with lower titres, especially in puppies.

### The significance of age

During our research, veterinary officials from the regional agriculture development commissionership recommended vaccinating puppies younger than three months old. Large-scale studies indicate that older dogs that have likely received vaccinations at least once in their lives, have a greater immune response, as confirmed by our research [19–22]. It appears that vaccination of puppies under three months old can only be recommended if a booster vaccination can be provided one month later and the vaccination certificate is given at that time to avoid false security for the owner because the next vaccination can only be carried out one year later.

Our findings are in accordance with those of Koutchoukali et al. [23], who demonstrated that older dogs have better titres than younger ones. This might be because adult dogs have stronger immune systems than puppies [24]. Moreover, titres seen in our study are similar to those observed in free-roaming African and Asian dogs, as well as in puppies studied by Morters [25, 26], since their antibody dynamics are comparable.

### Booster vaccination

Even if the vaccines pass the potency test, their effectiveness in sustaining a strong immune response for a year could be affected by field conditions. In our study, among all dogs, 48% remained sero-positive until D180 but only 7% of previously sero-negative dogs seroconverted on D30 and kept sero-positive until D365 post-immunisation. These findings are congruent with those of Minke et al. [27], who observed that 120 days post-immunisation with Nobivac Rabies vaccine, 7% of previously sero-negative dogs remained sero-positive. Future studies should be undertaken in Tunisia to compare different vaccines under the same field conditions.

These results indicate that, at least with the vaccine used in this study, a single vaccine is inadequate, especially for initial vaccination. Several studies [28–32] corroborate this viewpoint and suggest that an additional vaccine dose may be required, especially during the first year of life. In contrast, Darkaoui's research in Morocco [33] indicates that a single dose is adequate to achieve successful immunisation under conditions comparable to those observed in Tunisia. As with past vaccine effectiveness trials conducted in Tunisia, however, rigorous steps were taken in that study to safeguard the cold chain and adhere to the manufacturer's recommendations. Our study is distinct by the fact that it was performed under Tunisian vaccination campaign conditions to demonstrate the reality in the field.

Possible explanations for the dogs sero-positive at D0 failing to maintain seropositivity at D30 include inappropriate vaccine administration on D30 or ineffective vaccine potency.

Dogs being sero-negative for an entire year and exceeding the seropositivity threshold after a vaccination booster could be explained by the interaction of cellular immunity or by the fact that, even if they do not reach the seropositivity threshold, a weak immunological memory can result in an effective booster vaccination response.

It is agreed that rabies can be eradicated if 70% of the canine population is vaccinated [5–7]. It appears that in the Tunisian context the vaccination coverage is below this number. We confirm that in this study, vaccination under current field conditions, even with minimal facilities, allows seronconversion. This helps us to establish that, as mentioned in the literature [34–36], mass vaccination campaigns are successful. Indeed, vaccination induces an immune response even in the absence of a sufficient cold chain, meaning that the increase in rabies cases in Tunisia is not necessarily attributable to a failure of the vaccination process. It is presumed that the total number of dogs has increased over the years, but this has not been accounted for in the vaccination coverage rate estimate. Thus, the increase in animal rabies cases could be linked to an inaccurate estimate of the dog population, and therefore to lower coverage than reported. Over the years it has been established, at least in Tunisia that the increase in cases of animal rabies undoubtedly leads to an increase in the risk of the appearance of human rabies [37]. Increasing public awareness and improving vaccination campaign logistics are the two most important strategies to affect the epidemiology of rabies and eliminate the disease in order to save human and animal lives.

## Conclusion

In most circumstances, the current rabies vaccination protocol triggers an immunological response in field conditions in Tunisia. The results of this investigation show that primary immunisation does not guarantee seropositivity for a full year and that seropositivity is maintained in older dogs that had received multiple vaccinations previously. A booster vaccine is thus recommended 1 to 3 months after the primary vaccination.

## Methods

### Study site

This study was undertaken in Tunisia (North Africa) during the start of the 2017 national rabies vaccination campaign, which was administered via door-to-door and central-point immunisation programs by the regional agricultural development commissionership's veterinary staff.

**Fig. 6:**
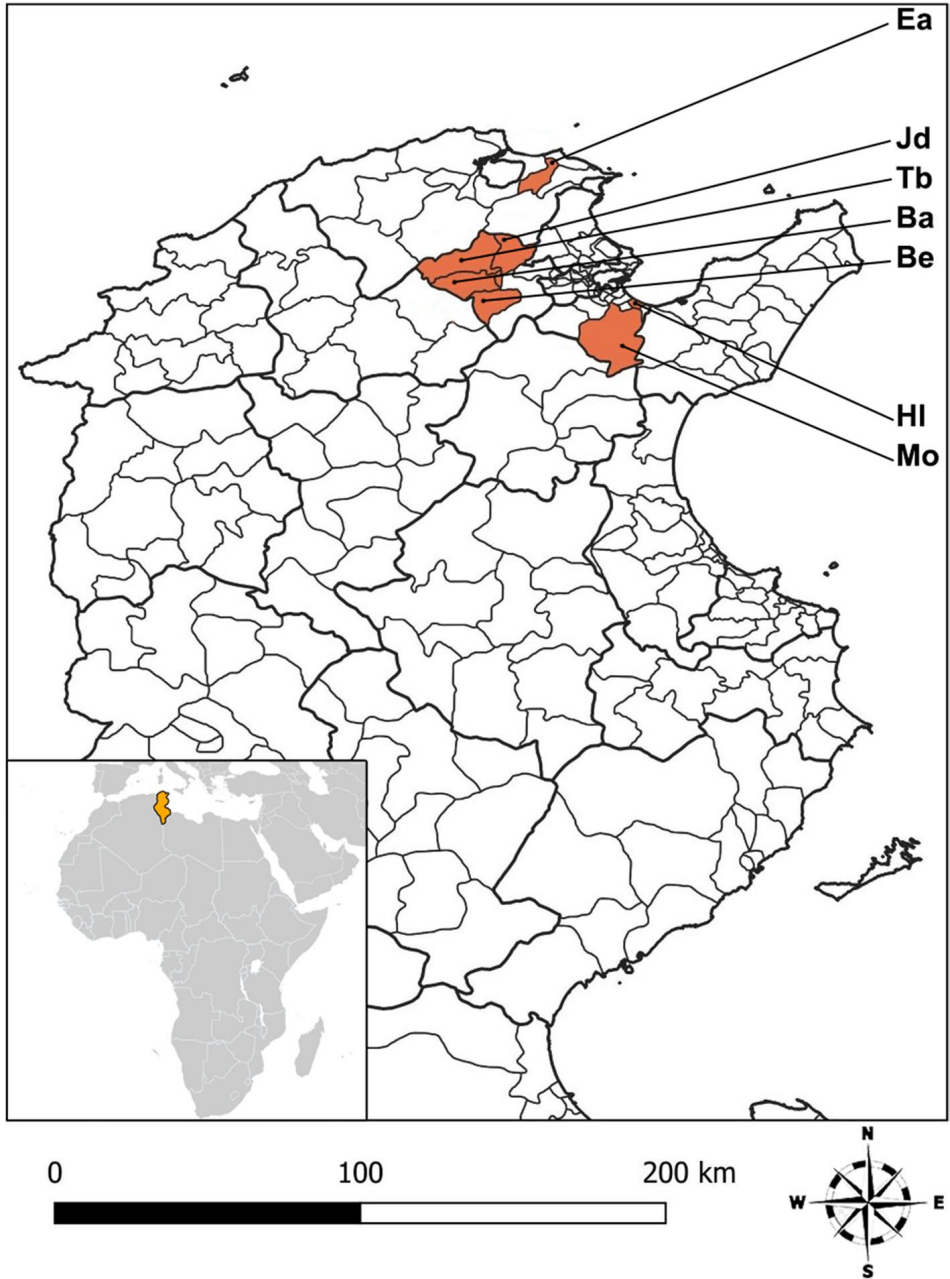
Study sites in Tunisia. Investigations were conducted in seven municipalities colored in orange that represent three governorates including rural, semi-urban and urban areas. Abbreviations: Ba: Battan, Be: Borj el ameri, Ea: El alia, Hl: Hammam lif, Jd: Jdaida, Mo: Mornag, Tb**:** Tborba

Ben arous, Bizerte, and Manouba governorates were selected for the investigation because they are endemic for rabies in different susceptible animal species (2, 61, and 15 cases, respectively) [10]. In addition, their socio-geographic settings differ (rural, semi-urban, and urban environments). In addition, their proximity to the laboratory facilitated the efficient and rapid collection of sera. Hammam lif (Hl) and Mornag (Mo) (Ben arous governorate), El alia (Ea) (Bizerte governorate), and Borj el ameri (Be), Battan (Ba), Tborba (Tb), and Jdaida (Jd) (Manouba governorate) were selected based on the vaccination team's permission to participate in the study (Fig. 6).

### Population assessment

After receiving informed consent, dog owners were asked to fill out two questionnaires: one about the home and one about the dog (Additional files 1 and 2). Each household's GPS coordinates were logged with a smartphone (using Google Maps) and linked to the dog owner's phone number to facilitate the next visit. We also noted the number of household members, the number of owned animals, and the species of those animals.

Before vaccination, dogs were identified using a photo and a colored polystyrene tape strip fastened with two rivets at neck level. The dog's coat color, size, gender, and approximate age (based on teeth inspection and owner declaration) were documented.

### Vaccination campaign

Vaccination campaigns were mostly conducted through door-to-door visits. Central-point vaccination was performed only in El alia and Mornag municipalities, according to veterinary team's preparedness.

Vaccines were stored using classical cool boxes and refrigerated cooling devices with ice packs. Temperature inside these different devices was monitored using a thermo-button that recorded the temperature every 15 min. Thermotrack software (ProgesPlus) was used to collect data. Weather reports provided environmental temperatures. The veterinary staff was given a questionnaire addressing various aspects of cold chain and vaccination campaign logistics (Additional file 3).

Nobivac Rabies (MSD animal health), an inactivated rabies vaccine comprising at least 2 IU of Rabies virus strain Pasteur RV per dosage, 0.15 ml/ml of aluminum phosphate, and 0.01 percent thiomersal was used. Before distribution to vets, each vaccination batch was tested under National Drug Control Laboratory's procedures. Each dog received 1 ml dosage subcutaneously using dosing injection guns (Hauptner-Herberholz) made of stainless steel with a capacity of 30 ml and adjustable dosing with reusable 13G metal needles.

### Sample collection, titration and analysis

Owners who agreed to conduct the procedure handled their dogs and gently turned the dog's face laterally. If the dog became agitated, we had to use a lasso. We avoided sampling if we considered the dog was at risk of biting. Blood was drawn from the cephalic vein and dogs were inoculated as described above. Blood samples were taken on days 0, 30, 90, 180, and 365 after immunisation. Serum was separated by centrifugation no later than the next day at 1500 rpm for six minutes and kept at -40 °C until further analysis. We opted to analyse sera of dogs from households visited four and five times.

In the Rabies laboratory of Pasteur Institute of Tunis, a total number of 364 sera from 51 dogs examined four times and 32 dogs examined five times were titrated using the Fluorescent Antibody Virus Neutralisation test, as recommended by the WOAH [38], WHO [39], and Cliquet et al. [40]. The bio-rad anti-Rabies Nucleocapsid Conjugate was employed in the research for staining. To eliminate internal variance, sera from each dog were titrated in the same series.

Dogs with titre greater than or equal to 0.5 IU/ml are referred to as "sero-positive". “Seroconversion” is defined as the rabies neutralising antibody titre reaching or exceeding the seropositivity threshold in response to immunisation.

### Statistical analysis

Factors that potentially influence immune response (previous immune status, age, sex, cold chain control, presence of ectoparasites and nutrition) were individually statistically compared and examined using student test on logD50 values. An analysis of variance (ANOVA) was also used to check if these different factors had a significant effect on immune response in a multivariable analysis. This statistical analysis was performed using R (3.1.2) software.

## Supplementary Information

**Additional file 1** Datasheet 1: Household questionnaire.

**Additional file 2** Datasheet 2: Dog questionnaire.

**Additional file 3** Datasheet 3: Veterinarian questionnaire.

## Data Availability

Most data generated or analysed during this study are included in this article. The other data are available from the corresponding author on request.

## Abbreviations

C: Celsius degree
D: Day
FAVN: Fluorescent Antibody Virus Neutralisation
GMT: Geometric Mean Titre
GPS: Global Positioning System
IU/ml: International Units per millilitre
kg: Kilogram
WOAH: World Organisation for Animal Health
rpm: Rotation per minute
WHO: World Health Organisation
