# Supplementary material for: Evaluation of the immune response of dogs after a mass vaccination campaign against rabies in Tunisia
Source: BMC Vet Res. 2023 Jan 30;19:24. doi: 10.1186/s12917-023-03582-8 (PMC9885660; doi:10.1186/s12917-023-03582-8)
Supplement: Supplementary file 1 — Additional file 1 Datasheet 1: Household questionnaire. [file 12917_2023_3582_MOESM1_ESM.docx]

**HOUSEHOLD QUESTIONNAIRE**

| \|  \|  \|  \|  \| \| --- \| --- \| --- \| --- \|  \|  \|  \|  \| \| --- \| --- \| --- \|   **Data Sheet N° Date of the visit:**    **Number of household residents :**  **Total number of animals :**  **Number of dogs: Vaccinated: yes number: no number:**  **Number of cats: Vaccinated: yes number: no number:**  **Presence of other susceptible animals : yes number : no** |
| --- | --- | --- | --- | --- | --- | --- | --- |
