# Supplementary material for: Evaluation of the immune response of dogs after a mass vaccination campaign against rabies in Tunisia
Source: BMC Vet Res. 2023 Jan 30;19:24. doi: 10.1186/s12917-023-03582-8 (PMC9885660; doi:10.1186/s12917-023-03582-8)
Supplement: Supplementary file 2 — Additional file 2 Datasheet 2: Dog questionnaire. [file 12917_2023_3582_MOESM2_ESM.doc]

**DOG QUESTIONNAIRE**

| **Date of rabies vaccination:**  **Data Sheet N°**   |  |  |  |  | | --- | --- | --- | --- |     **Name of dog owner :**  **Photo**   |  |  |  | | --- | --- | --- |   **Address:**  **Phone number:**  **GPS coordinates longitude: latitude:**  **Size: Big Small Coat color :**  **Dog’s markings:** 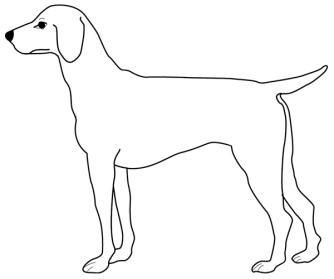 **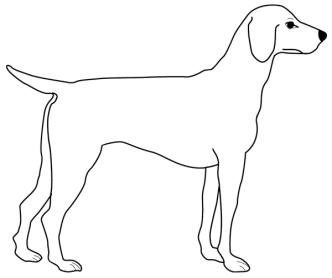**  left flank right flank  **Age: ≤2 months [3-5] months [6-12] months not estimated**  **Reason(s) age is not estimated:**  **Dog’s gender: M F**  **District of origin:**      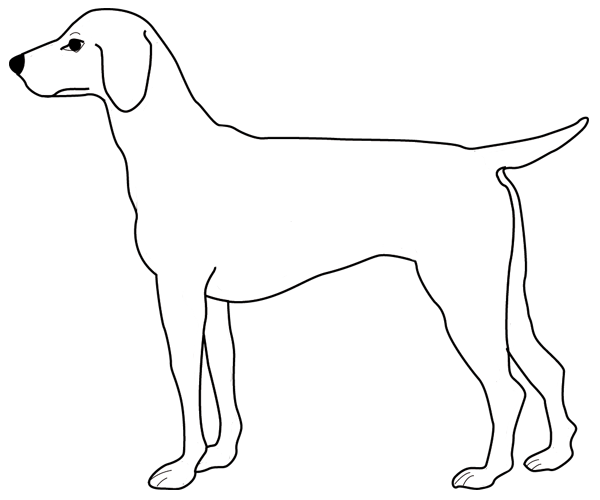  **Site of vaccine’s injection:** *(on the drawing)*  **Quality of the injection : good bad**  **Was the animal previously vaccinated? no yes**  **If yes, date of the vaccination :**  **Use of treatment against external parasites: no yes frequency :**  **Presence of external parasites/observable skin lesions: no yes**  **Use of dewormer against internal parasite: no yes frequency :**  **Type of food:** |
| --- | --- | --- | --- | --- | --- | --- | --- |
