# Supplementary material for: Evaluation of the immune response of dogs after a mass vaccination campaign against rabies in Tunisia
Source: BMC Vet Res. 2023 Jan 30;19:24. doi: 10.1186/s12917-023-03582-8 (PMC9885660; doi:10.1186/s12917-023-03582-8)
Supplement: Supplementary file 3 — Additional file 3 Datasheet 3: Veterinarian questionnaire. [file 12917_2023_3582_MOESM3_ESM.doc]

### VETERINAIRIAN QUESTIONNAIRE

| **Data sheet N° Date:**   |  |  | | --- | --- |  |  |  |  |  | | --- | --- | --- | --- |   **Equipment for the storage of vaccine stocks**  **Cold room Refrigerator Other :**  **Equipment temperature (depending on the personnel in charge of vaccine storage)**  **>0°C +4°C >10°C No answer**  **Temperature (measured by the team at the start of each field trip):**  **Injection route:**  **IM SC Other :**  **Needle change frequency:**  **Use of cooler: yes no**  **Use of ice packs: yes no**  **Vehicle availability :**  **available full time available depending on the planning not available**  **Number of staff available:**  **0 1 2 3 >3** |
| --- | --- | --- | --- | --- | --- | --- |
